# Ytterbium-Modified Rapidly Solidified Mg-Zn-Ca Alloys: Improvements in Strength and Corrosion Resistance for Biodegradable Implant Applications

**DOI:** 10.3390/ma18214959

**Published:** 2025-10-30

**Authors:** Zuzana Molčanová, Beáta Ballóková, Karel Saksl, Miroslav Džupon, Dóra Zalka, Zoltán Dankházi

**Affiliations:** 1 Institute of Materials Research of Slovak Academy of Sciences, Watsonova 47, 040 01 Košice, Slovakia; molcanova@saske.sk (Z.M.); ksaksl@saske.sk (K.S.); mdzupon@saske.sk (M.D.); dzalka@saske.sk (D.Z.); 2 Institute of Materials, Faculty of Materials, Metallurgy and Recycling, Technical University of Košice, Letná 1/9, 042 00 Košice, Slovakia; 3 Department of Materials Physics, Eötvös Loránd University, Pázmány Péter Sétany 1/a, 1117 Budapest, Hungary; zoltan.dankhazi@ttk.elte.hu

**Keywords:** magnesium alloys, biodegradable alloys, rapid solidification, mechanical properties, corrosion resistance, microstructure

## Abstract

**Highlights:**

**What are the main findings?**

- Yb addition (2–4 at.%) enhances amorphous formation in Mg_66_Zn_30_Ca_4_ alloys.
- Yb addition improved mechanical and electrochemical properties of Mg-Zn-Ca al-loys.

**What are the implications of the main findings?**

- Controlled Yb incorporation optimize structural integrity and biocompatibility in Mg–Zn–Ca–Yb systems.
- Yb modification enhances the suitability of Mg-Zn-Ca alloys for biodegradable or-thopedic implants.

**Abstract:**

Biodegradable alloys are increasingly studied in medical research to find the best balance between mechanical performance and biocompatibility. Magnesium (Mg) alloys are particularly attractive because of their low density, good mechanical properties, and compatibility with the human body. However, rapid corrosion limits their wider use. Recently, Mg alloys containing zinc (Zn) and calcium (Ca) have drawn interest due to more suitable degradation rates. In this study, Mg_66_Zn_30_Ca_4_ was chosen as the base alloy due to its high mechanical strength, excellent biocompatibility, and favorable corrosion behavior, making it an ideal starting point for biodegradable implants. We investigated how adding ytterbium (Yb) affects the mechanical and corrosion behavior of this base alloy. Mg_66−x_Zn_30_Ca_4_Yb_x_ (x = 2, 4, 6) rods were produced using rapid solidification, and their mechanical and electrochemical properties were systematically evaluated. The results show that adding Yb significantly improves corrosion resistance while maintaining high mechanical strength, making these materials promising for applications requiring both strength and controlled degradation. Corrosion tests indicated that the surface of the samples developed an oxygen-rich layer approximately 18 μm thick, as identified by EDS mapping. Overall, Yb modification enhances the suitability of Mg-Zn-Ca alloys for biodegradable orthopedic implants.

## 1. Introduction

Traditional bone implants are typically made from non-degradable biomaterials, designed for either temporary or permanent use. While effective in many cases, these materials often require secondary surgeries for removal or replacement, increasing health risks and medical costs for patients [1,2,3]. An emerging and more sustainable alternative is the use of biodegradable implants, which are engineered to gradually dissolve within the body after fulfilling their intended function. These implants not only eliminate the need for secondary procedures, but also produce degradation products that can promote tissue regeneration and enhance overall healing [4]. Among the candidate materials for biodegradable implants, magnesium (Mg) and its alloys have attracted significant attention due to their low density (~1.74 g∙cm^−3^), elastic modulus close to that of natural bone, favorable mechanical properties, and excellent biocompatibility [5,6,7]. However, crystalline Mg alloys face critical limitations, including rapid corrosion, hydrogen gas evolution, localized alkalization, and a degradation rate faster than natural tissue healing [8,9]. These factors compromise implant performance and limit clinical adoption. To address these challenges, researchers have increasingly focused on magnesium-based bulk metallic glasses (Mg-BMGs), a relatively novel class of materials characterized by their amorphous atomic structure and superior properties compared to crystalline alloys [10,11,12]. Unlike conventional Mg alloys, Mg-BMGs exhibit:

- high yield strength (>300 MPa) and enhanced toughness,
- lower elastic modulus, closer to that of cortical bone, minimizing stress shielding,
- better corrosion resistance and improved biocompatibility.

Mg-BMGs are produced through rapid quenching techniques—such as injection casting, melt spinning, and tilt casting—that suppress atomic rearrangement and “freeze” the disordered atomic structure, resulting in a stable amorphous phase. This unique structure allows Mg-BMGs to incorporate high concentrations of alloying elements without forming undesired crystalline phases, enabling better control over mechanical and corrosion behavior [13,14,15]. The development of Mg-BMGs began with the pioneering work of Inoue et al. [16], who synthesized Mg–Cu–Y-based amorphous alloys with excellent glass-forming ability (GFA). Since then, advances have been made by tailoring alloy compositions through elemental substitution or microalloying strategies to improve glass formation, corrosion resistance, and biological performance [17,18,19].

To address the limitations of traditional magnesium alloys in biomedical applications, researchers have increasingly focused on incorporating non-toxic alloying elements that offer both biocompatibility and functional benefits. Elements such as zinc (Zn) and calcium (Ca) have shown strong potential due to their biological significance and synergistic effects: Zn contributes to immune function and mitigates impurity-induced corrosion, while Ca plays a key role in promoting osteogenesis and accelerating bone regeneration [19,20]. Building on this approach, recent studies have also explored the use of rare earth (RE) doping, particularly ytterbium (Yb), which has been shown to enhance corrosion resistance, improve antibacterial performance, and improve mechanical stability of magnesium-based implants [18]. Yb is considered particularly attractive among RE elements because of its relatively low toxicity and favorable biocompatibility profile. In addition, Yb can participate in the formation of stable intermetallic phases, which contribute to a more homogeneous microstructure and reduce localized corrosion sites. Moreover, the incorporation of Yb has been reported to increase glass-forming ability in Mg-based metallic glasses, thereby further improving their structural reliability under physiological conditions.

In this study, we focus on enhancing mechanical and corrosion properties of Mg-Zn-Ca alloys through Yb addition. The Mg-Zn-Ca-Yb system was chosen for its combination of low density, high specific strength, good biocompatibility, and controlled degradation, making it a strong candidate for biodegradable implants and lightweight structural components [21,22,23]. Specifically, Mg_66_Zn_30_Ca_4_ was selected as the base alloy because it already has high mechanical strength, excellent biocompatibility, and suitable corrosion behavior, providing a solid foundation for further improvement via Yb modification. Its well-known baseline properties allow us to systematically study the effect of Yb on structural stability and corrosion resistance.

The main goal of this study is to determine whether adding Yb can further improve both the mechanical performance and electrochemical stability of the base Mg_66_Zn_30_Ca_4_ alloy. 

## 2. Materials and Methods

### 2.1. Materials Preparation

The experimental alloys were synthesized from high-purity metals: calcium (Ca, 99.5%, Thermo Scientific, Waltham, MA, USA), magnesium (Mg, 99.98%, Alfa Aesar, Ward Hill, MA, USA), zinc (Zn, 99.99%, Alfa Aesar, Ward Hill, MA, USA), and ytterbium (Yb, 99.9%, Alfa Aesar, Ward Hill, MA, USA). The elemental metals were compacted into 13 mm diameter pellets using a hydraulic press. The compacted charge (total mass: 5 g) was placed in a graphite tube and loaded into a Melt Spinner SC equipped with a casting setup. The metals were inductively heated to 670 °C and held for 5 min to ensure complete homogenization of the melt. The process was carried out under an argon atmosphere to prevent oxidation. The molten alloy was then injected through a small nozzle at the bottom of the graphite tube into a water-cooled cylindrical copper mold using argon overpressure. The resulting rod-shaped ingots had a diameter of 3 mm and a length of approximately 130 mm and were used for further experiments. This rapid solidification method allowed the preparation of alloys with varying Yb content and uniform microstructure.

### 2.2. Microstructure and Chemical Characterization

For microstructure characterization, the samples were cross-sectioned and embedded in conductive powder. They were then ground on SiC papers and polished with 1 μm diamond paste. Finally, the samples were etched to reveal the microstructure. Microstructure and chemical composition were analyzed by scanning electron microscopy (SEM; TESCAN VEGA 3 LMU, TESCAN, Brno, Czech Republic) equipped with an energy dispersive spectrometer (EDS). The chemical composition of cast alloys is in Table 1. Slight deviations were observed between nominal and experimentally measured compositions. Additionally, the prepared samples exhibited good homogeneity throughout the entire volume of the ingot. 

Volumetric mass density of the studied samples was determined by Archimedes’ principle. Measurements were performed in pure ethanol with a density of 0.7856 g∙cm^−3^ by an analytical balance Kern ABT 120-4M with a special density determination kit ABT-A01. Powder (non-compacted) samples were analyzed by X-ray diffraction (XRD; Philips Xpert Pro, Almelo, The Netherlands) applying CuKα radiation.

### 2.3. Mechanical Testing

Samples for compression tests were cut from the cast ingots into cylindrical shapes with a diameter of 3 mm and length of 6 mm. Compressive tests were performed along the casting direction using a universal testing machine (TiraTest 2300, TIRA GmbH, Schalkau, Germany). Ultimate compressive strength measurements were also conducted at room temperature on the same machine. Hardness and elastic modulus were determined using the depth-sensing indentation (DSI) technique, which involves applying a controlled load while simultaneously measuring the penetration depth of the indenter into the material with high precision (<0.1 nm). The tests were conducted on a TTX-NHT nanoindentation device (CSM Instruments) equipped with a Berkovich diamond tip operating in linear loading mode. A maximum load of 150 mN was applied with a holding time of 10 s. The resulting load–penetration (P–h) curves were analyzed following the Oliver and Pharr [24] method to obtain hardness and elastic modulus as functions of indentation depth, along with the elastic and plastic deformation energies. Up to 20 indentations were performed per sample and statistically evaluated.

### 2.4. Electrochemical Properties

The electrochemical behavior of the magnesium alloys was evaluated by potentiodynamic polarization in Hank’s solution at 22.5 °C and 37 °C. Before recording the polarization curves, the electrodes have been stabilized in the solution for 15 min. A conventional three-electrode configuration was used, consisting of a saturated calomel electrode (SCE) as the reference, a platinum counter electrode, and the magnesium alloy sample as the working electrode. Electrochemical measurements were performed using a VoltaLab PGZ402 Potentiostat (Hach Lange, Lognes, France) at a scan rate of 1 mV∙s^−1^. Corrosion current density (*I_corr_*) was determined using the Tafel extrapolation method.

## 3. Results and Discussion

### 3.1. Microstructure Characterization

**Figure 1:**
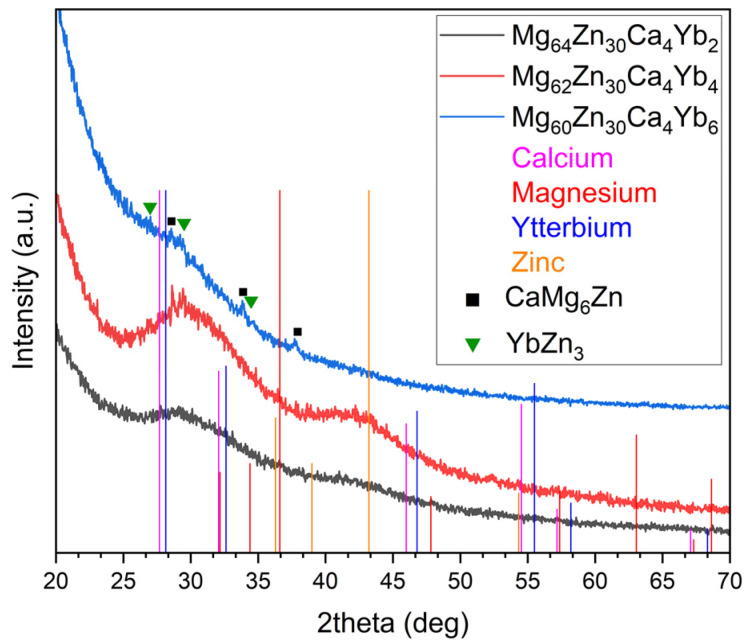
XRD patterns of the cast Mg_66−x_Zn_30_Ca_4_Yb_x_ (x = 2, 4, 6 at %) alloys.

XRD patterns of Mg-Zn-Ca-Yb alloys (Figure 1) reveal broad diffuse maxima between 20° and 50° 2θ, confirming their predominantly amorphous nature. Reference lines for pure Ca, Mg, Yb, and Zn show no significant overlap with the measured peaks, indicating the absence of detectable crystalline elemental phases in the bulk alloys. Minor sharp peaks in Mg_62_Zn_30_Ca_4_Yb_4_ likely originate from surface oxidation or trace impurities rather than bulk crystallinity. At the highest Yb content (Mg_60_Zn_30_Ca_4_Yb_6_), XRD indicates an increased fraction of intermetallic phases (CaMg_6_Zn and YbZn_3_), consistent with the formation of a more complex multiphase structure. These results suggest that Yb additions up to 4 at. % enhance amorphous-forming ability, whereas higher concentrations favor crystallization and development of a finely structured multiphase alloy. Excessive Yb thus promotes nucleation and growth of thermodynamically stable crystalline phases, limiting further amorphous formation.

**Figure 2:**
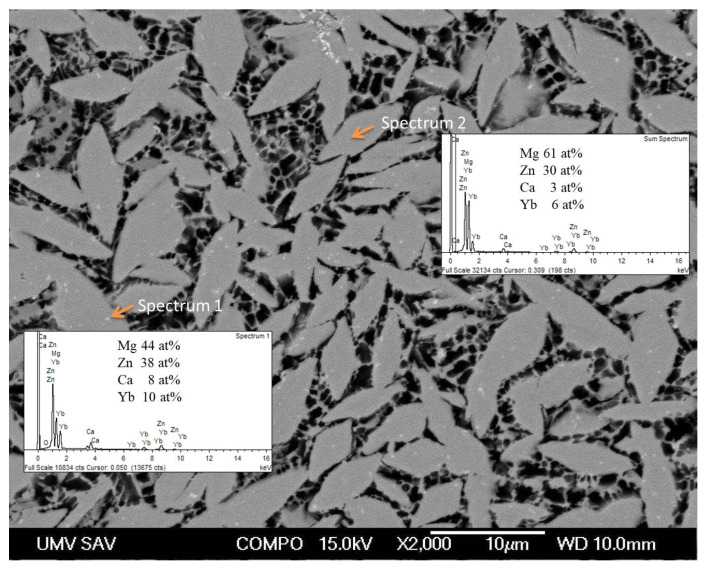
The microstructure of Mg_60_Zn_30_Ca_4_Yb_6_ alloy prepared by rapid solidification.

The SEM micrograph of Mg_60_Zn_30_Ca_4_Yb_6_ (Figure 2) is consistent with XRD results, showing a fine, densely packed arrangement of plate-like intermetallics embedded in a refined Mg–Zn matrix.

EDS measurements reveal that the bright regions are highly enriched in Zn, Ca, and Yb, whereas the darker matrix remains Mg-dominant with limited Yb solubility. This compositional partitioning signifies that higher Yb concentrations promote solute segregation and intermetallic phase stability during rapid solidification.

### 3.2. Mechanical Properties

The mechanical properties of the investigated Mg-Zn-Ca-Yb alloys are summarized in Table 2, including density, compressive ultimate strength, nanoindentation hardness (H_IT_), and elastic modulus (E_IT_). For comparative reference, values for Ti_6_Al_4_V alloy (in the field of orthopedics, it is one of the most commonly used alloys) and human cortical bone are also provided. The measured densities of the magnesium-based alloys ranged from 3.177 ± 0.016 g∙cm^−3^ for Mg_64_Zn_30_Ca_4_Yb_2_ to 3.46 ± 0.012 g∙cm^−3^ for Mg_60_Zn_30_Ca_4_Yb_6_. These densities are notably lower than that of the Ti_6_Al_4_V alloy (4.42 g∙cm^−3^), providing a potential advantage in reducing implant weight, while remaining higher than the typical density of human cortical bone (1.7–2.1 g∙cm^−3^). In terms of compressive ultimate strength, Mg_62_Zn_30_Ca_4_Yb_4_ achieved the highest measured value at 591 MPa, followed by Mg_64_Zn_30_Ca_4_Yb_2_ at 528 MPa, and Mg_60_Zn_30_Ca_4_Yb_6_ at 518 MPa. Although these values do not reach the compressive strength of Ti_6_Al_4_V (850 MPa), they substantially exceed the compressive strength of human cortical bone (100–230 MPa [25]), supporting their suitability for temporary load-bearing applications. Nanoindentation testing revealed H_IT_ values of 3.26 ± 0.165 GPa for Mg_64_Zn_30_Ca_4_Yb_2_, 3.49 ± 0.028 GPa for Mg_62_Zn_30_Ca_4_Yb_4_, and a reduced hardness of 2.88 ± 0.321 GPa for Mg_60_Zn_30_Ca_4_Yb_6_. These hardness values are considerably higher than those of human bone (between 0.40 and 0.90 GPa [26,27]), but remain below that of the Ti_6_Al_4_V reference alloy (5.6 GPa). The elastic modulus (E_IT_) of the Mg-Zn-Ca-Yb alloys remained within a relatively narrow range, from 70 to 72 GPa. This modulus is significantly lower than that of Ti_6_Al_4_V (120 GPa), but much closer to the elastic modulus of human bone (17–30 GPa). A closer match to the bone modulus is highly desirable, as it helps minimize stress shielding effects and promotes more uniform load transfer to the surrounding biological tissue.

### 3.3. Electrochemical Characteristics

**Figure 3:**
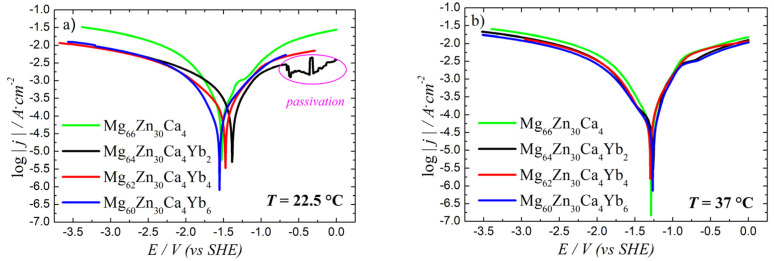
Tafel plots of samples measured at (**a**) 22.5 °C and (**b**) 37 °C, respectively.

Potentiodynamic polarization tests were performed in Hank’s solution at the scan rate 1 mV∙s^−1^ using a three-electrode configuration (saturated calomel electrode as reference, platinum counter electrode). The polarization curves were evaluated from Tafel plots (Figure 3) and were based on the ASTMG59-97 standard [28] procedure (Equation (1)):

(1) $$v=\frac{{Kj}_{corr}EW}{\rho }$$

where v is the corrosion rate (in mm/year) and K is 3270 for units of (mm/year, μA/cm^2^, g/eq, g/cm^3^). $j_{corr}$ is the electrochemical corrosion current density (μA∙cm^−2^), *EW* is the equivalent weight (g/eq) and *ρ* is the density (g∙cm^−3^) of the Mg-based alloys. The corrosion behavior of the Mg-Zn-Ca-Yb alloys was systematically investigated using Tafel analysis at two temperatures, 22.5 °C and 37 °C, to simulate room and body temperature conditions, respectively. The measured parameters included corrosion current (*I_corr_*), corrosion current density (*j_corr_*), polarization resistance (*R_corr_*), corrosion rate (*v*), and corrosion potential (*E_corr_*), as summarized in Table 3.

At 22.5 °C, the Mg_64_Zn_30_Ca_4_Yb_2_ alloy exhibited the highest corrosion current (4.03 mA) and the highest corrosion rate (18.95 mm/y), suggesting the most rapid degradation among the tested compositions. The corrosion potential for this alloy was measured at −1.16 V, while its polarization resistance was 77 Ω. With increasing ytterbium content to 4 at.% (Mg_62_Zn_30_Ca_4_Yb_4_), a marked reduction in the corrosion current (1.07 mA) and corrosion rate (7.94 mm/y) was observed, along with a more negative corrosion potential of −1.34 V and a slightly higher polarization resistance of 82 Ω. Further increasing the ytterbium content to 6 at.% (Mg_60_Zn_30_Ca_4_Yb_6_) resulted in the lowest corrosion current (1.01 mA) and the lowest corrosion rate (5.22 mm/y), with a polarization resistance of 83 Ω and a corrosion potential of −1.37 V. The Mg_60_Zn_30_Ca_4_Yb_6_ sample exhibited passivation behavior at 22.5 °C (Figure 3). These results suggest that increasing ytterbium content generally improves the corrosion resistance of the alloy under room temperature conditions.

At 37 °C, which is closer to physiological conditions, the corrosion rates decreased compared to room temperature, indicating a stabilization of the corrosion process. The Yb-containing alloys show reduced corrosion rates compared to baseline, but the improvement is less dramatic than at 22.5 °C (values converge). The Mg_64_Zn_30_Ca_4_Yb_2_ alloy showed a corrosion current of 1.55 mA and a corrosion rate of 6.83 mm/y, with a corrosion potential of −1.07 V and polarization resistance of 72.9 Ω. The Mg_62_Zn_30_Ca_4_Yb_4_ alloy demonstrated a comparable corrosion current of 1.44 mA and a corrosion rate of 6.73 mm/y, with a more negative corrosion potential (−1.17 V) and higher polarization resistance (79.7 Ω). The Mg_60_Zn_30_Ca_4_Yb_6_ alloy presented the lowest corrosion current at 1.28 mA and the lowest corrosion rate of 6.07 mm/y, with a corrosion potential of −1.14 V and the highest polarization resistance among all compositions at 94.8 Ω.

At room temperature, this indicates that Yb stabilizes protective corrosion films, most likely through the formation of Yb_2_O_3_/Yb(OH)_3_, which limits anodic dissolution. The corrosion potential also shifted to slightly more negative with Yb addition, consistent with altered cathodic kinetics. At 37 °C, higher solution conductivity and accelerated film breakdown reduce the protective effect of the Yb_2_O_3_/Yb(OH)_3_ [29].

These results clearly demonstrate that increasing Yb content improves corrosion resistance, with Mg_60_Zn_30_Ca_4_Yb_6_ consistently performing best at both temperatures. The slight decrease in corrosion rates at 37 °C for all alloys indicates a mild stabilization of the corrosion process under physiological conditions, and the relative ranking of corrosion resistance among the alloys remains consistent.

**Figure 4:**
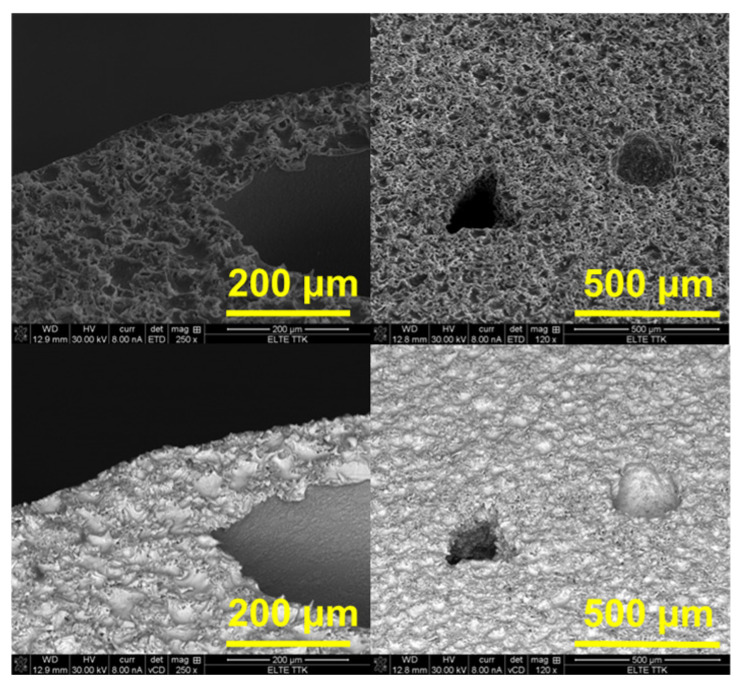
Micrographs of the prepared samples before the corrosion process.

Figure 4 shows SEM micrographs before the corrosion process. The micrographs display the surface morphology and cross-sectional features of a porous material.

**Figure 5:**
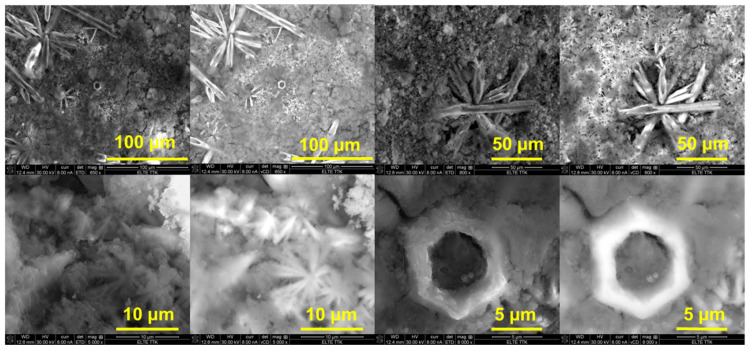
Micrographs of the prepared samples after the corrosion process.

The micrographs in Figure 5 show the surface morphology of samples after corrosion testing. The surfaces exhibit localized corrosion with pit formation and crystalline corrosion products. Needle- or flower-like structures are visible, suggesting the formation of corrosion products, possibly Mg(OH)_2_ or complex zinc-calcium hydroxides. Several pits appear in the higher magnification images, with a hexagonal or rounded shape, typical of micro-galvanic corrosion in Mg alloys.

**Figure 6:**
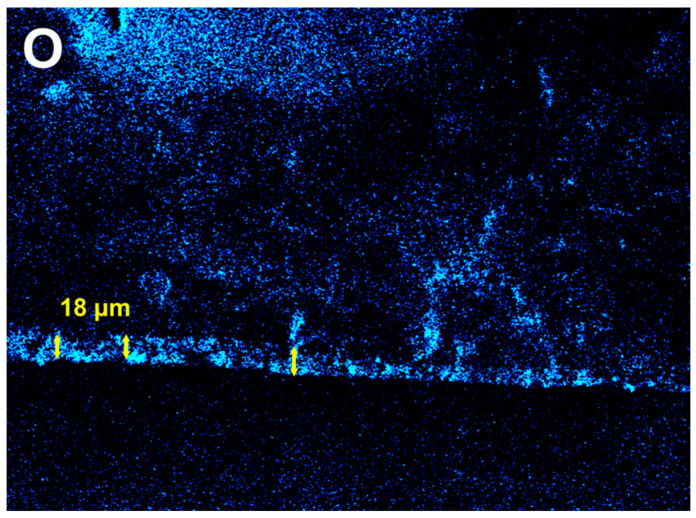
EDS elemental mapping of oxygen (O) in the cross-section of the sample.

Figure 6 presents the oxygen (O) elemental mapping of the sample’s cross-section. A distinct oxygen-enriched layer with an average thickness of approximately 18 µm is clearly visible near the surface. This continuous oxygen-rich region indicates the formation of a surface oxide layer. The relatively uniform thickness suggests a homogeneous oxidation process across the examined area rather than localized pitting corrosion.

## 4. Conclusions

In this study, the effect of Ytterbium addition on rapidly cooled Mg_66_Zn_30_Ca_4_ metallic glass was investigated to improve mechanical properties and corrosion resistance.

The present results show the ability of alloys in the Mg-Zn-Ca system to form amorphous structures with an ingot diameter of 3 mm by adding a low concentration of ytterbium.

According to the XRD analysis, the alloys containing 2 and 4 at.% Yb (Mg_64_Zn_30_Ca_4_Yb_2_ and Mg_62_Zn_30_Ca_4_Yb_4_) exhibit broad diffuse halos, characteristic of predominantly amorphous structures, indicating that these compositions possess a high glass-forming ability under rapid solidification conditions. In contrast, the Mg_60_Zn_30_Ca_4_Yb_6_ alloy displays small sharp diffraction peaks, confirming a partially crystalline microstructure. This transition from amorphous to crystalline structure with increasing Yb content demonstrates that the Yb concentration strongly influences the solidification pathway and structural stability of the alloy. These findings are in good agreement with the statement reported in the literature [30], which highlights that the highest amorphous-forming ability occurs at around 4 at.% Yb. Both studies identify this composition as a critical point where Yb optimally disrupts atomic packing, suppresses long-range ordering, and minimizes atomic clustering, thereby favoring amorphous formation.

Mechanical testing confirms that the Mg-Zn-Ca-Yb alloy exhibits a favorable balance of properties, including an intermediate elastic modulus, sufficient compressive strength, and reduced density relative to traditional metallic biomaterials. These characteristics align closely with the mechanical behavior of human bone, thereby minimizing the risk of modulus mismatch and subsequent stress shielding. Yb-modified alloys exhibit higher strength than human cortical bone. Their compressive strength ranges from 528 to 591 MPa, which is more favorable than that of commercially available titanium biomaterials (850 MPa). Also, the nanohardness and indentation modulus values of Yb-modified alloys were closer to those of human bone than the commercially available Ti_6_Al_4_V alloy.

Electrochemical studies demonstrate that increasing Yb content in the Mg-Zn-Ca alloy significantly enhances corrosion resistance. This improvement is evidenced by decreased corrosion rates, increased polarization resistance, and more negative corrosion potentials, all of which are indicative of enhanced electrochemical stability. Specifically, the addition of ytterbium (Yb) promotes the formation of a more stable oxide layer, which limits anodic dissolution and improves corrosion resistance, as reported in a previous study on Mg-based alloys [31]. Notably, corrosion rates measured at physiological temperature (37 °C) are consistently lower than those at room temperature (22.5 °C), suggesting a stabilization of the corrosion process under conditions closer to the human body. These findings align with the results of studies showing that Yb-containing alloys exhibit reduced corrosion rates at 37 °C, with the corrosion behavior stabilizing under physiological conditions.

In addition to its influence on the structural evolution, the incorporation of Yb has also been associated with biological performance improvements in Mg-based alloys. Some studies [32,33] have reported that the addition of ytterbium to magnesium alloys not only supports osteoblast adhesion and proliferation but also provides effective antibacterial protection, making it highly attractive for biomedical applications. The live/dead cell assay conducted in these studies confirmed the excellent cytocompatibility of Yb-containing Mg extrusion alloys, particularly for compositions containing around 1.0 at.% Yb, where enhanced cell viability was attributed to improved corrosion resistance imparted by trace Yb addition. These findings align with the present results, which show that Yb addition significantly affects the alloy’s microstructure and phase stability, suggesting that controlled Yb incorporation could simultaneously optimize both structural integrity and biocompatibility in Mg–Zn–Ca–Yb systems.

Collectively, these findings substantiate that the addition of ytterbium to Mg-Zn-Ca alloys optimizes their microstructural and electrochemical properties, rendering them promising candidates for future biodegradable orthopedic implants that combine mechanical strength, corrosion resistance, and biocompatibility.

## Figures and Tables

**Table 1:** Chemical composition of the cast Mg_66−x_Zn_30_Ca_4_Yb_x_ (x = 2, 4, 6 at %) alloys.

Sample	Mg	Zn	Ca	Yb
Mg_64_Zn_30_Ca_4_Yb_2_	63	31	4	2
Mg_62_Zn_30_Ca_4_Yb_4_	62	30	4	4
Mg_60_Zn_30_Ca_4_Yb_6_	59	31	4	6

**Table 2:** Comparison of the mechanical properties of the investigated Mg-Zn-Ca-Yb alloys with the commercially used Ti_6_Al_4_V alloy and human bone.

	*ρ* (g·cm^−3^)	UCS (MPa)	H_IT_ (GPa)	E_IT_ (GPa)
Mg_64_Zn_30_Ca_4_Yb_2_	3.177 ± 0.016	528	3.26 ± 0.165	71
Mg_62_Zn_30_Ca_4_Yb_4_	3.22 ± 0.017	591	3.49 ± 0.028	70
Mg_60_Zn_30_Ca_4_Yb_6_	3.46 ± 0.012	518	2.88 ± 0.321	72
Ti_6_Al_4_V	4.42	850	5.6	120
Human Bones	1.7–2.1	70–280	0.82 ± 0.092	17–30

**Table 3:** Electrochemical parameters obtained from Tafel analysis.

Results of the Tafel Analysis at 22.5 °C					
Alloys	*I*_corr_ (mA)	*j*_corr_ (µA∙cm^−2^)	*R*_corr_ (Ω)	*v* (mm/y)	*E*_corr_ (V)
Mg_66_Zn_30_Ca_4_	9.54	2004	27	41.3	−1.24
Mg_64_Zn_30_Ca_4_Yb_2_	4.03	915	77	18.9	−1.16
Mg_62_Zn_30_Ca_4_Yb_4_	1.07	362	82	7.9	−1.34
Mg_60_Zn_30_Ca_4_Yb_6_	1.01	234	83	5.22	−1.37
**Results of the Tafel Analysis at 37 °C**					
**Alloys**	***I*_corr_** **(mA)**	***j*_corr_** **(µA∙cm^−2^)**	***R*_corr_** **(Ω)**	***v*** **(mm/y)**	***E*_corr_** **(V)**
Mg_66_Zn_30_Ca_4_	2.32	494	57	10.0	−1.11
Mg_64_Zn_30_Ca_4_Yb_2_	1.55	329	72.9	6.8	−1.07
Mg_62_Zn_30_Ca_4_Yb_4_	1.44	306	79.7	6.73	−1.17
Mg_60_Zn_30_Ca_4_Yb_6_	1.28	273	94.8	6.1	−1.14

## Data Availability

The original contributions presented in this study are included in the article. Further inquiries can be directed to the corresponding author.

## Abbreviations

The following abbreviations are used in this manuscript:

UCS	Ultimate compressive strength
EDS	Energy dispersive spectrometer
BMG	Bulk metallic glasses
E_IT_	Indentation elastic modulus
H_IT_	Indentation hardness
*ρ*	Density
*I_corr_*	Corrosion current
*j_corr_*	Corrosion current density
*R_corr_*	Polarization resistance
*v*	Corrosion rate
*E_corr_*	Corrosion potential
